# Outbreak of unusual H_2_S-negative monophasic *Salmonella* Typhimurium strain likely associated with small tomatoes, Sweden, August to October 2019

**DOI:** 10.2807/1560-7917.ES.2019.24.47.1900643

**Published:** 2019-11-21

**Authors:** Soledad Colombe, Cecilia Jernberg, Emma Löf, Anna Lindqvist Angervall, Henrik Mellström-Dahlgren, Leif Dotevall, Malin Bengnér, Ingela Hall, Lena Sundqvist, Sharon Kühlmann-Berenzon, Ilias Galanis, Mats Lindblad, Anette Hansen, Moa Rehn

**Affiliations:** 1 Public Health Agency of Sweden, Solna, Sweden; 2 European Programme for Intervention Epidemiology Training (EPIET), European Centre for Disease Prevention and Control (ECDC), Solna, Sweden; 3 County Council Department of Communicable Disease Control and Prevention, Region Västra Götaland, Gothenburg, Sweden; 4 County Council Department of Communicable Disease Control and Prevention, Region Jönköping, Jönköping, Sweden; 5 Swedish Food Agency, Uppsala, Sweden

**Keywords:** disease outbreaks, epidemiology, Salmonella infections, WGS, whole genome sequencing, Sweden

## Abstract

Sweden is investigating an outbreak of monophasic *Salmonella* Typhimurium. Eighty-two nationally-distributed cases have been confirmed, with date of symptom onset between 28 August and 29 October. Cases were 51 years of age on average (range: 0–89) and the majority of cases were female (62%). A case–control study was conducted and suggested small tomatoes as source of the outbreak (adjusted odds ratio (OR): 10.8, 95% confidence interval (CI): 4.15-112.68, p value < 0.001), and a trace-back investigation led to a single, non-Swedish producer in Europe. Both the *Salmonella* strain and the source of the outbreak are rarely encountered in Europe. Results from investigation at the producer are pending.

## Background

Salmonellosis is one of the most common food-borne pathogen in the European Union (EU), with 90,000 cases reported annually [1]. In Sweden, clinical and laboratory diagnoses of *Salmonella* infection are notifiable by law. Around 2,000 cases are reported annually, the majority being infected abroad [2]. Sweden has *Salmonella* control programs in place for feed, animals and food products of animal origin, and Swedish meat and eggs are generally free from *Salmonella* [3].

Monophasic *Salmonella* Typhimurium is among the most common serovars in Europe, including in Sweden, with sequence type (ST)34 being the most common sequence type [4]. It is historically known to be commonly transmitted by pork products [4]. Although incubation time can be 6 to 72 hours for *Salmonella*, it has been shown that the median incubation period range for 95% of *Salmonella* Typhimurium outbreaks is 12 to 192 hours [5].

## Outbreak detection

On 10 September 2019, the County Council Department of Communicable Disease Control and Prevention (CDC-department) in Jönköping informed the Public Health Agency of Sweden (PHAS) of the detection of five domestic cases of *Salmonella* with no obvious epidemiological connection over the 2 days prior. This was not only an uncommon accumulation of cases, but all had also been caused by an unusual *Salmonella* strain phenotypically shown to be hydrogen sulfide (H_2_S) negative on traditional growth agar medium, i.e. the characteristic black pigmentation of the colonies was lacking. Then on 11 September 2019, the CDC-department in Västra Götaland reported a sixfold increase in domestic *Salmonella* group B cases compared with the same time period over the last 4 years. On 12 September, a small cluster of seven monophasic *Salmonella* Typhimurium ST3478 was identified among isolates from across the country. Isolates were identified as part of the routine microbial surveillance program where all isolates of *Salmonella* from domestic infection are sent to PHAS for further typing using whole genome sequencing (WGS). On 17 September, the cluster had grown to 27 isolates and an outbreak was declared at the national level, with an investigation initiated to describe the outbreak and identify the source of infection. The outbreak team included PHAS, the Swedish Food Agency (SFA), the CDC-departments and the municipalities’ environmental health departments. Eighty-two nationally-distributed cases were confirmed, with date of symptom onset between 28 August and 29 October. The following report describes the outbreak investigation until 18 November 2019.

## Methods

### Outbreak case definition

A suspected case was defined as a case with a laboratory result matching the unusual phenotype of the *Salmonella* strain, infected in Sweden according to the clinician, and notified after 15 August 2019 since the earliest reported date of symptom onset was 28 August 2019. A case was confirmed if it belonged to the outbreak WGS cluster of monophasic *Salmonella* Typhimurium ST3478.

### Epidemiological investigation

Data on age, sex, county of residence, date of symptom onset and sample date were collected via the national system for notifiable diseases. Date of symptom onset was replaced by sample date if date of symptom onset was missing. An initial trawling questionnaire, set for a recall period of 7 days and asking general questions about the consumption of foods known to be potential sources for *Salmonella,* was sent to cases by email. If cases did not have an email address or were too sick to access the online questionnaire, it was administered on paper via regular mail or by telephone interviews.

### Case–control study

We further investigated the source of the outbreak by conducting a case–control study. From a national random pool of controls (n = 5,900) available at PHAS [6], we selected 384 controls. These controls were matched on age group, sex and county of residence to each of the first 48 cases of the outbreak (8 controls per case). Some of the first 48 cases of the outbreak never filled in the case–control questionnaire. Since more cases were confirmed after the start of the case–control study, new cases were also included in the analysis if they had answered the case–control questionnaire. A general description of the potential source of the outbreak was made public to address public concern on 7 October 2019 and cases confirmed after that date were not included in the study. In total, 45 of 71 confirmed cases up to 7 October 2019 answered the case–control questionnaire and were thus included in the analysis, regardless if they had initially been matched to controls or not. Only 29 of 45 cases were matched to controls, and as the number of matched case control pairs was not sufficient to perform a matched analysis, we conducted an unmatched analysis. Controls were excluded if they had travelled abroad during the recall period or reported symptoms compatible with salmonellosis, i.e. diarrhoea and either of fever, abdominal pain or bloody diarrhoea.

The case–control questionnaire that was sent to both cases and controls included those food items from the trawling questionnaire that had been consumed by more than 40% of the cases, and asked if the consumed vegetables and fruits were organic or non-organic, and details about retail stores they did their general grocery shopping at. The recall period was set to 7 days before symptom onset (cases) or before answering the questionnaire (controls).

We conducted unmatched Firth logistic regression [7] adjusting for age group and sex, both when including individual exposures in the model as well as when including multiple exposures. We calculated odds ratios (OR), their 95% confidence interval (95% CI) and p values for models including individual exposures, and adjusted odds ratios (adjOR), 95% CI and p values for models including multiple exposures. The latter were built by step-wise backward selection, starting with a full model including all items with an exposure among cases > 40%, a p value < 0.2 and an OR >1 in the individual exposure model, and removing food items one-by-one based on Akaike information criterion (AIC) improvement. The threshold for significance was set at 0.05.

### Microbiological investigations of human isolates

The local clinical microbiological laboratories cultured and isolated *Salmonella* from submitted faecal samples. Colonies lacking the black pigmentation on Xylose-Lysine-Deoxycholate (XLD) and Deoxycholate-Citrate (DC) agar plates, i.e. H_2_S negative, leaving the colonies light pink, were defined as the suspected outbreak strain.

Multilocus sequence typing (MLST) at PHAS and subsequent ST was calculated by mapping the raw reads to a reference sequence for each loci of the EnteroBase *Salmonella* 7-gene MLST scheme. Single nt polymorphisms (SNPs) were called based on an assembly of one of the outbreak strains using CLC Assembly Cell version 4.4.2 (Qiagen Bioinformatics, Hilden, Germany), minimum 10x coverage, 90% read consensus. Minimum spanning trees were generated using MSTgold [8] and recombinations were filtered by looking for SNPs with a pairwise distance of 500 nt. One representative outbreak sequence was deposited to the European Nucleotide Archive (ENA) (https://www.ebi.ac.uk/ena), number ERR3577233. For the sequence submitted to ENA, the MiSeq instrument (Illumina, San Diego, California, United States) was used [9].

### Trace-back investigation and microbiological analysis of food

Based on the outcome of the epidemiological investigation, the SFA conducted a trace-back investigation of the food source and retail company. Information on the likely date of purchase was collected from seven cases with an onset of disease early in the outbreak, and the product was traced back to the producer.

Leftover suspected food source was collected from two cases and analysed for *Salmonella* spp. at commercial laboratories.

## Results

### Descriptive epidemiology

A total of 82 cases were notified and confirmed by WGS, with date of symptom onset between 28 August 2019 and 29 October 2019. Cases were distributed between 12 of 21 counties in Sweden, with the majority of cases occurring in the south-western part of the country (67%, n = 55). Cases had a mean age of 51 years (range: 0–89) and were mostly female (62%, n = 51).

**Figure 1:**
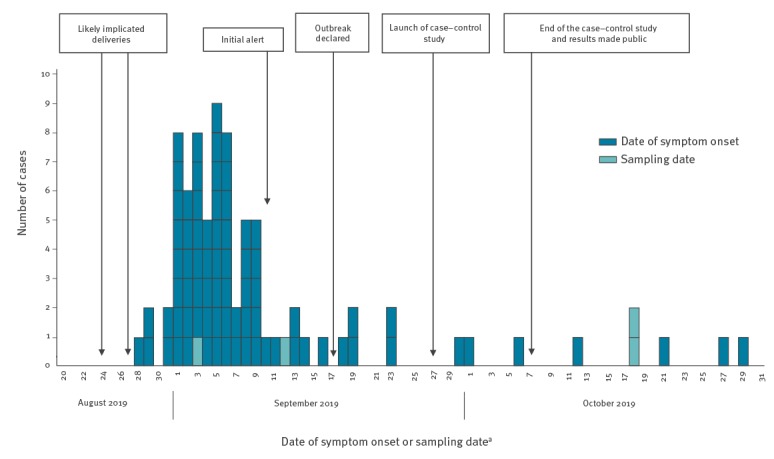
Number of confirmed cases, by date of symptom onset/sampling, timeline of the monophasic *Salmonella* Typhimurium outbreak investigation, Sweden, August–October 2019 (n = 82)

^a^ Date of symptom onset was replaced by sample date if date of symptom onset was missing.

The distribution of date of symptom onset suggested a point source outbreak, with a common food source having a short shelf-life (Figure 1).

A total of 40 cases (50%) responded to the trawling questionnaire. Of the 40, 38 reported having eaten tomatoes within the incubation period, and at least seven cases reported the same type and brand of tomatoes.

### Case–control study

Response rates to the case–control study questionnaires were 45 of 71 (63%) among cases and 328 of 384 (85%) among controls. Controls resided in the same counties as cases and one additional county, and had a mean age of 54 years (range: 1–88). Of controls, 62% (n = 202) were female. Logistic regressions for each of the 16 exposures (Table 1) showed that small tomatoes, grilled chicken from the store and shopping at retail stores belonging to Company A, a nation-wide food retailer owning different retail chains, were associated with being a case (Table 1). The final multivariable model included both small tomatoes (OR: 10.8, 95% CI: 4.15–112.68, p < 0.001, percentage exposed among cases: 98%) and grocery shopping at company A (OR: 8.5, 95% CI: 3.95–22.41, p < 0.001, percentage exposed among cases: 82%). The final model did not include grilled chicken as exposure among cases was low (31%).

**Table 1:** Percentage exposed among cases and controls, odds ratio and adjusted odds ratio of cases compared with controls in monophasic *Salmonella* Typhimurium outbreak investigation, Sweden, August–October 2019 (n = 45 cases)

Food item or retail company	Cases exposed (n = 45)^a^		Controls exposed (n = 328)^a^		Model for each exposure^b^			Final model^c^		
	n	%	n	%	OR	95% CI	p value	AdjOR	95% CI	p value
Small tomatoes	44	98	173	53	10.1	3.94–103.41	< 0.001	10.8	4.15–112.68	< 0.001
Grocery shopping at a retail store belonging to Company A	37	82	117	36	7.8	3.75–19.80	< 0.001	8.5	3.95–22.41	< 0.001
Grilled chicken from store^d^	14	31	31	10	2.9	1.67–4.93	< 0.001	NA	NA	NA
Nectarines	11	24	48	15	1.6	0.88–2.58	0.10	NA	NA	NA
Cucumbers	35	78	211	66	1.5	0.93–2.76	0.10	NA	NA	NA
Large tomatoes	26	58	147	45	1.4	0.92–2.27	0.11	NA	NA	NA
Grocery shopping at a retail store belonging to Company B	7	16	39	12	1.4	0.52–3.13	0.44	NA	NA	NA
Smoked ham	26	58	151	47	1.4	0.87–2.16	0.17	NA	NA	NA
Grapes	18	40	105	33	1.3	0.79–1.97	0.32	NA	NA	NA
Bell pepper	22	49	135	42	1.2	0.78–1.90	0.39	NA	NA	NA
Grocery shopping at a retail store belonging to Company C	14	31	103	31	1.0	0.49–1.94	0.97	NA	NA	NA
Grocery shopping at retail stores belonging to other companies	4	9	32	10	1.0	0.26–2.58	0.99	NA	NA	NA
Carrots	23	51	183	58	0.8	0.53–1.32	0.44	NA	NA	NA
Apples	16	36	145	46	0.7	0.46–1.17	0.20	NA	NA	NA
Grocery shopping at a retail store belonging to Company D	3	7	42	13	0.6	0.11–1.53	0.31	NA	NA	NA
Grocery shopping at a retail store belonging to Company E	25	56	242	74	0.4	0.23–0.85	0.01	NA	NA	NA

adjOR: adjusted odds ratio; CI: confidence interval; NA: not applicable; OR: odds ratio.

^a^ Only cases and controls responding to the case–control study questionnaires are included.

^b^ Each exposure adjusted for sex and age group (0–17 years, 18–59 years, 60 years and above).

^c^ Adjusted for sex, age group (0–17 years, 18–59 years, 60 years and above) and the one other exposure in the model.

^d^ The final model did not include grilled chicken as exposure among cases was low.

For those who had eaten small tomatoes (n = 44), small organic tomatoes and grocery shopping from Company A were associated with being a case (Table 2).

**Table 2:** Percentage exposed among cases and controls and adjusted odds ratio of exposures among those who ate small tomatoes in the monophasic *Salmonella* Typhimurium outbreak investigation, Sweden, August–October 2019 (n = 44 cases)

Exposure	Cases exposed (n = 44)		Controls exposed (n = 173)		adjOR^a^	95% CI	p value
	n	%	n	%			
Small organic tomatoes	26	59	47	27	2.5	1.22–6.14	0.017
Small non-organic tomatoes	22	50	111	64	1.3	0.59–3.05	0.56
Retail store belonging to Company A	37	84	57	33	9.0	3.87–28.91	< 0.001

adjOR: adjusted odds ratio; CI: confidence interval.

^a^ Adjusted for other exposures presented in the table, age group (0–17 years, 18–59 years and 60 years or above) and sex.

We further stratified the analysis on those that shopped from company A and those that did not. Among those who had eaten small tomatoes and bought their groceries from Company A (n = 37), small organic tomatoes (OR: 2.7, 95% CI: 1.23–7.20, p value 0.021) were associated with being a case, while small non-organic tomatoes were not (OR: 0.9, 95% CI: 0.36–2.17, p value 0.73). For those who did not shop from company A, interpretation was difficult because of a very small number of cases (n = 6): small non-organic tomatoes (OR: 7.90, 95% CI: 0.78–Infinite, p value 0.091) and small organic tomatoes (OR: 2.55, 95% CI: 0.26–17.53, p value 0.29).

### Descriptive microbiology of human isolates

**Figure 2:**
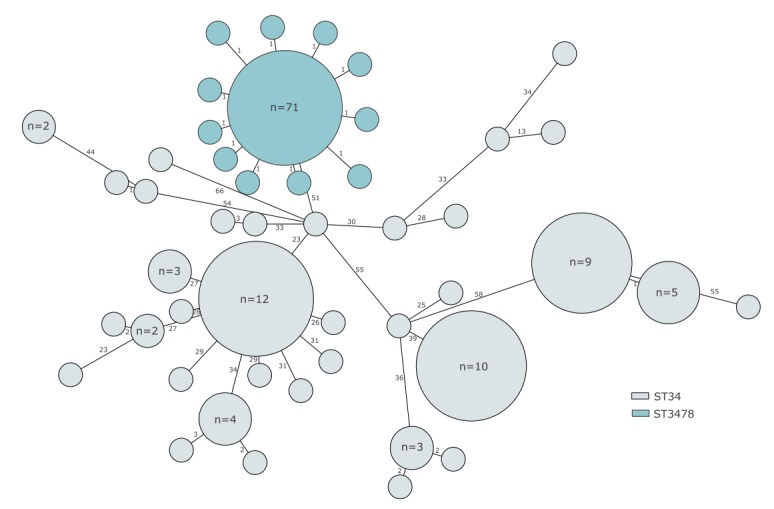
Minimum spanning tree of monophasic *Salmonella* Typhimurium isolates from domestic cases, Sweden, 1 January–31 October 2019

All isolates clustered with 0–2 SNP differences, and were identified as monophasic *Salmonella* Typhimurium, ST3478, which is similar to the more commonly encountered ST34 (Figure 2). All isolates were H_2_S negative, which is a rare phenotypic feature among *Salmonella* strains regardless of serotype.

The common strain of 76, ST34 is shown in grey while the outbreak strain, ST3478, is shown in blue. SNP differences are shown next to the branches. The length of the branches are not proportional to the SNP distances. The Minimum spanning tree was generated using MSTgold [2] and recombinations were filtered by looking for SNPs with a pairwise distance of 500 nt.

### Trace-back investigation and microbiological analysis of food

All small organic tomatoes sold at Company A’s retail stores were delivered by one wholesaler in Sweden and sold under the private label of Company A. Deliveries at the end of August and the beginning of September showed that the product was produced and packed by one company in an EU country. Small organic tomatoes bought by cases within a few days before symptom onset most likely originated from either or both of two deliveries arriving to Sweden on 24 and 27 August 2019. Products from these deliveries were sold across the country.

No leftover small tomatoes available from cases’ households were from the suspected deliveries. Two samples from other deliveries were nonetheless analysed for *Salmonella* spp., with negative results. No grilled chicken was tested for.

## Outbreak control measures

On 19 September, PHAS decided to inform all local clinical microbiological laboratories in Sweden about the unusual phenotypic characteristic of the outbreak strain to enable faster identification of cases and to ensure that the strain was not missed. On 7 October 2019, the possibility of small tomatoes being the source of the outbreak was published on PHAS’ website with general advice on how to prevent infection with *Salmonella*, and stating that the contaminated tomatoes were unlikely to still be on the market [10].

On 19 September, PHAS also contacted and shared a representative outbreak sequence (Ion Torrent data) with public health institutes in Denmark, Finland, Norway, neighbouring countries with an awareness of challenges regarding comparing IonTorrent data and Illumina data, and the United Kingdom, which does SNP analysis on a routine basis for an initial sequence comparison. None of these countries found a matching sequence. On 3 October 2019, an urgent inquiry (UI-603) was posted on the European Centre for Disease Prevention and Control (ECDC) Epidemic Intelligence Information System for Food and Waterborne Diseases and Zoonoses (EPIS-FWD), to enquire about cases in other European countries. According to responses to the urgent inquiry in EPIS from nine countries until 21 October 2019, this specific ST had only been seen in the past in only a few sporadic cases.

On 11 October, the SFA informed the competent authority in the country of the producer of the small organic tomatoes and the results of the follow up are pending.

No recall was performed because tomatoes from the implicated deliveries were no longer on the market.

## Discussion

This is the first reported outbreak of *Salmonella* in Sweden with small tomatoes as the likely source of infection and only the second in Europe [11], despite tomatoes being a well-known source of *Salmonella* outbreaks in the United States [12-14]. The results from this investigation highlight the importance of considering vegetables as a possible vector of pathogens traditionally thought to be associated with animal products [15,16]. Despite not being able to sample the implicated batches of tomatoes for analysis of *Salmonella*, the epidemiological link to small tomatoes was strong. Samples from potential cases are still being analysed by WGS. Two more cases were confirmed on 13 November 2019 and investigation regarding their exposures is ongoing.

The main analysis pointed towards small tomatoes as the likely source. Our subset analysis suggested that small organic tomatoes specifically might be the main source, but organic tomatoes only explained 60% of cases. This discrepancy could be explained by recall bias or by contamination of small non-organic tomatoes at the producer. Local media coverage after the start of the case–control study could have also biased the late answers of controls, reducing the estimate of the OR.

Grilled chicken was not considered to be a true risk factor for being a case in our outbreak as the exposure among cases was low. In addition, the chicken is grilled directly in the supermarket and it would be unlikely that supermarkets from across the country undercooked and improperly stored their grilled chicken over the same time period.

ST3478 has been rarely seen in Europe thus far, yet we observed an outbreak with over 80 cases. We can only speculate on the reasons why Sweden was the sole country affected by this outbreak. One possibility could be that just a few batches were contaminated and all of them were sent to Sweden. Another possibility is that because the strain lacked the black pigmentation on traditional growth agar medium, it could have been missed in other countries. Countries should be aware that this ST in combination with its unusual phenotypic feature (H_2_S-negative) could go unnoticed on traditional growth agar medium because of the lack of black pigmentation. It underlines the importance of a close collaboration between clinical laboratories, food laboratories and offices for control of communicable diseases in identifying and investigating outbreaks.
